# Is FAME 2 a breakthrough for PCI in stable coronary disease?

**DOI:** 10.1007/s00392-014-0801-4

**Published:** 2014-12-06

**Authors:** Udo Sechtem

**Affiliations:** Abteilung für Kardiologie, Robert-Bosch-Krankenhaus, Auerbachstr. 110, 70376 Stuttgart, Germany

In the Fractional Flow Reserve versus Angiography for Multivessel Evaluation 2 (FAME 2) study, 888 patients with stable coronary artery disease were randomised either to fractional flow reserve (FFR)-guided percutaneous coronary intervention (PCI) plus optimal medical therapy or optimal medical therapy alone [1]. Before randomization, FFR was measured in all angiographically visible stenoses and patients who had at least one stenosis in a major coronary artery with a FFR of 0.80 or less were randomised. Patients who did not fulfil criteria were followed in a separate registry. This inclusion algorithm resulted in an optimal selection of patients for PCI as judged by current standards. Recruitment was halted prematurely because of a significant between-group difference in the percentage of patients who had a primary endpoint event. The primary endpoint was a composite of death, myocardial infarction (MI) or urgent revascularization. The difference in primary endpoint events at 1 year into the study (12.7 % in the medical-therapy group vs. 4.3 % in the PCI group) was driven by a higher number of urgent revascularizations in the medical-therapy group (11.1 vs. 1.6 %). There was also a trend towards fewer hard events (death or myocardial infarction) in the PCI group (3.4 vs. 3.9 % or 15 vs. 17 events, respectively) [1]. In a so-called “landmark” analysis from 8 days to 1 year (which excluded the excess number of peri-interventional endpoints in the PCI group occurring between days 1 and 7), this small difference was transformed into a hazard ratio of 0.42 in favour of PCI with a *p* value of 0.053.

The design of this important study included two aspects which resulted in a disadvantage for the medical-therapy group. First, patients had to be informed about the fact that they had a significant stenosis which was left untreated because they had been randomised to the medical-therapy group. It is easy to imagine what kind of effect such knowledge would have if some even mild form of chest pain recurred. The patient would necessarily interpret this as a signal of impending doom and present to the hospital. There he would be treated as a patient with unstable angina and—knowing that he harboured a high-grade stenosis—be immediately brought to the catheterization laboratory. The treating doctor would not hesitate and dilate the stenosis. This would generate an endpoint “urgent revascularisation” in the medical-therapy group. Unfortunately, it would be difficult to design the study in a way that such an effect could be avoided. Only if the medically treated group would undergo a sham procedure [2]—similar to the design of the simplicity-3 trial [3]—would it have been possible to achieve comparable and unbiased conditions. Is the occurrence of chest pain, be it provoked by exercise or occurring at rest, an emergency which needs to be treated by PCI immediately? Resting chest pain occurs frequently in patients with stable angina and is often caused by coronary spasm [4]. One very good treatment for spasm is the addition of a calcium antagonist of the existing medication. However, we are not told that this was ever tried in the medical-therapy patients. Thus, the study design inevitably puts patients in the medical-therapy group at a higher risk for the occurrence of events. This first bias involved in the study design makes it necessary to focus on the smaller group of patients who presented with an unstable coronary syndrome and objective evidence of ischemia such a new troponin elevation or ST-segment changes not present in the previous ECG. At 1 year such an ACS constellation was present in 23 (5.2 %) vs. 4 (0.9 %) patients in the medical-therapy and the PCI groups indicating that there was a real difference in truly indicated urgent revascularisations. These patients, however, represent just half of the total number of urgent revascularizations [49 (11.1 %) vs. 7 (1.6 %)] in the medical-therapy and PCI groups, respectively.

The second disadvantage for the medically treated group was due to the fact that the larger number of revascularizations in the medical-therapy group in patients without objective evidence of ischemia probably also triggered additional hard events in the medical-therapy group. PCI not infrequently results in periprocedural elevations of troponin. Such an event, however, was likely counted as an additional hard event (non-ST-elevation myocardial infarction) occurring in the group with medical therapy. The authors do not comment on how such events were counted and on the number of events occurring this way.

Recently, FAME 2 again produced headlines when the 2-year follow-up results were simultaneously presented at the ESC Congress in Barcelona and online in the New England Journal of Medicine [5]. At 2 years, the “landmark” analysis of hard events (death or MI) occurring after day 7 following randomisation and PCI up to 2 years produced a significantly higher number of events in the medical-therapy group than in the PCI group. The excitement was heightened by the fact that this was the first trial of PCI vs. medical therapy ever which showed a reduction of hard events in the interventional group. Less exciting was that the significant difference in the number of primary outcomes in favour of the PCI group already demonstrated after 1 year was unchanged at 2 years (8.1 vs. 19.5 % in the PCI vs. the medical-therapy group). As after 1 year, this difference was exclusively driven by a lower rate of urgent revascularizations in the PCI group (4.0 vs. 16.3 %). And again, only a subgroup of patients had their revascularizations triggered by objective markers of ischemia such as troponin rises or ischemic changes on electrocardiography. However, as after 1 year such revascularisations were significantly less frequent in the PCI group than in the medical-therapy group (3.4 vs. 7 %, *p* = 0.01). Interestingly, this “hard” between-group difference had become smaller at 2 years than after 1 year (3.6 vs. 4.3 %). There was no significant between-group difference in the total rates of death and MI (PCI group: 29 patients experiencing death or MI (6.5 %) vs medical group: death or MI in 36 patients (8.2 %, *p* = 0.37). The absolute number of events was 6 deaths in the PCI group and 8 deaths in the medical-therapy group. However, in each group, only 3 of the deaths were cardiac in origin. The number of MIs at the end of year 2 was 26 in the PCI group and 30 in the medical-therapy group (Fig. 1). This would include patients who had some troponin rise after urgent revascularization disfavouring the medical-therapy group (see above). Thus, one can also conclude that the trial clearly shows an absence of benefit in terms of hard events of well-conducted state-of-the-art PCI in stable angina patients. Interestingly, no graph (neither in the paper nor in the appendix) shows the total number of hard events over time including those occurring between days 0 and 7.

**Fig. 1:**
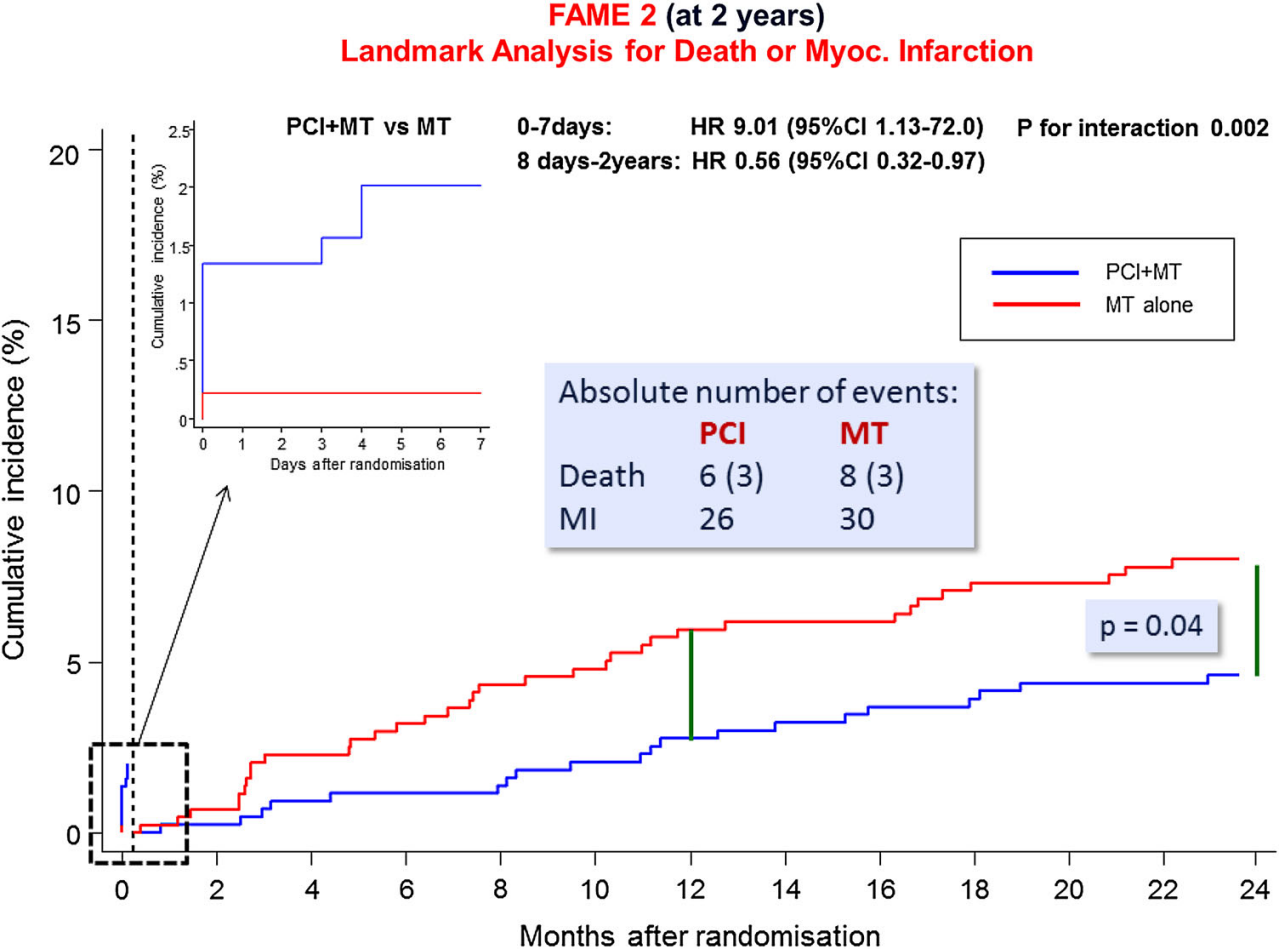
Kaplan–Meier Curve for the Death and Myocardial Infarction Landmark Analysis (adapted from [5]). The cumulative incidences of death or myocardial infarction in the two study groups, stratified on the basis of a landmark point at 7 days after randomization (*vertical dashed line*) are shown. The hazard ratios shown for PCI versus medical therapy were calculated separately for events that occurred within 7 days and those that occurred between 8 days and the end of follow-up at 2 years. Data for the first 7 days are not included in the period after 7 days. The *insets* show the data for days 0 to 7 on an expanded *y* axis. Hazard ratios below 1.00 denote a lower incidence of the end point in the PCI group than in the medical-therapy group. The absolute number of hard events is shown in *box*, the centre of the figure. *p* value denotes the significant difference calculated in favour of PCI in the 2-year landmark analysis. The *green bars* indicated the absolute difference between the two curves at 1 year. It is evident that at 2 years no further separation of the two curves occurred

Instead, attention was focused on the significantly lower rate in the PCI group when the first 7 days were excluded (4.6 vs. 8.0 %, *p* = 0.04). This skewed vision of the results was promoted by the fact that the lack of a significant difference in hard endpoints over the entire time from randomisation up to 2 years (which is only clinically meaningful) was placed in a table in the appendix whereas the “landmark” analysis was mentioned in the summary of the trial. Heartwire commented that “PCI is superior to medical therapy for the reduction of hard clinical endpoints in patients with stable coronary artery disease”. Such a disinformation policy will most likely create inaccurate brain engrams in readers who necessarily do not have the time to go into much detail when trying to keep abreast with current studies.

How can it be explained that despite the absence of an increasing difference between groups from year 1 to year 2 statistics of the “landmark” analysis came up with a significant difference in favour of the PCI group at year 2 which was not found after year 1? This effect was probably caused by the slightly larger number of total events in both groups whereas the absolute difference in both groups remained even decreased slightly. If one looks closer at the numbers of “urgent” revascularisations one sees that the difference in revascularizations triggered by objective evidence narrowed from 19 events at 1 year to 16 events at the end of the second year of follow-up indicating that during year two of follow-up both groups fared equally well. However, this is not mentioned anywhere in the paper.

Thus, a number of critical questions have to be asked. First, is it clinically important whether a *p* value changes from 0.053 to 0.04? Second, how many of the MIs in the two groups of FAME 2 were clinically relevant Q-wave MIs and how many were just mild troponin rises? Third, how many of these troponins rise occurred in the context of more or less urgent revascularization? Finally, can the slightly higher rate of deaths of all causes be used as an argument against medical therapy when the number of cardiac deaths was identical?

An argument in favour of immediate PCI in patients with stable angina who are FFR positive put forward by the authors was that more than 40 % of patients treated by medical therapy had crossed over by 2 years of follow-up, i.e. had undergone any revascularization (Fig. 2). However, another way of looking at the data (Fig. 3) is that almost 110 % of the patients in the PCI group had undergone any revascularization over the period of the 2 years whereas only slightly more than 40 % of those originally assigned to medical therapy alone had needed PCI. This means that more than 60 % of patients in the medical-therapy group had not needed PCI. If one puts the total number of revascularizations into one graph with the number of hard events one sees the discrepancy between difference in the numbers of revascularizations and the difference in the numbers of heart events. Considering that many of the hard events were probably mild troponin elevations following resting angina without objective evidence of ischemia, the achievement of PCI appears even less impressive.

**Fig. 2:**
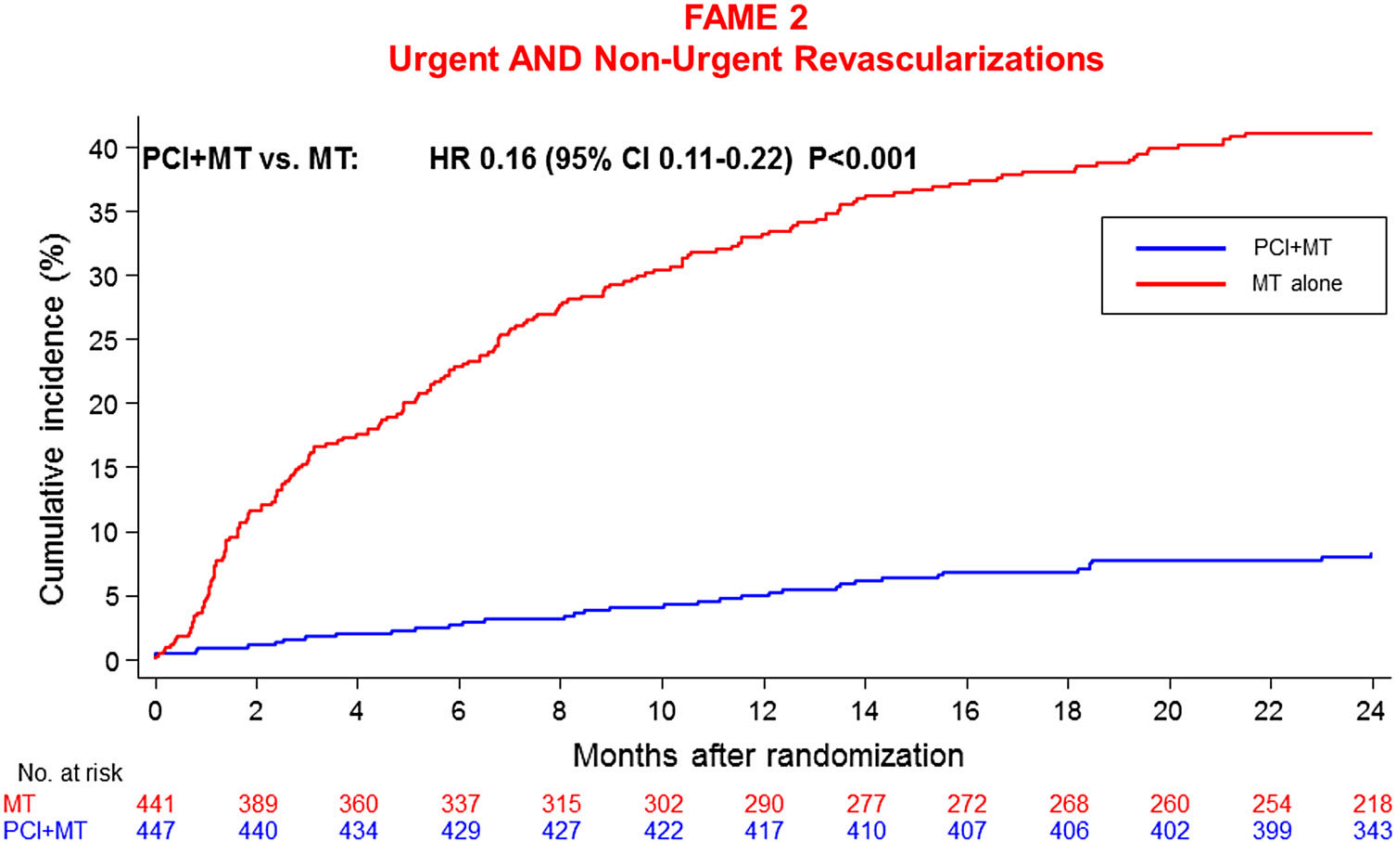
Urgent and non-urgent revascularisations in FAME 2 at 2 years. After 2 years, >40 % of patients treated by MT had crossed over, i.e. had undergone any revascularisation

**Fig. 3:**
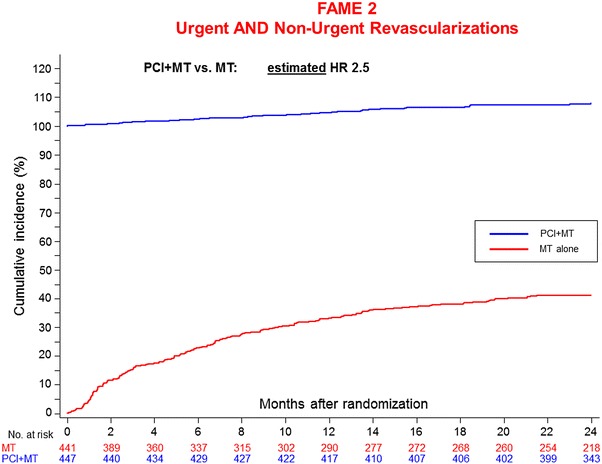
Modification of Fig. 2 with all points on the *blue curve* increased by 100 % revascularisation performed in that group. It becomes clear that the PCI procedure could be avoided in a large portion of patients in the medically treated group (about 60 %) without a worse outcome in terms of hard events

Thus, the main messages from the 2-year follow-up of the milestone FAME 2 study are the following:

*First* Immediate PCI in a cohort of patients in whom two-thirds have proximal or mid LAD stenosis and who have stable angina, does not result in a worse outcome than medical therapy alone. However, this statement is probably only true if PCI is FFR guided. *Second* The benefit in terms of reductions of myocardial infarction and death in the PCI group is very small and non-significant (1.7 %, *p* = 0.37). The difference does only seem to be statistically significant if one ignores the higher rate of MIs and death in the PCI group within the first 7 days after the intervention. However, this “landmark” type of analysis is clinically irrelevant. *Third* Repeated attacks of angina are of course worrying for the patient and reducing the number of patients with such symptoms is a valuable goal. However, revascularization of all the patients fulfilling the entire criteria for FAME 2 will result in overtreating many of these patients at unnecessary cost to society. Fourth: FAME 2 clearly demonstrates that no harm will be done if revascularization is postponed until the patient has reoccurrence of symptoms with or without some troponin rise.

The interventional community should not be enticed by the irrelevant yet highly publicised result of the “landmark” analysis to forget current guideline recommendations for the treatment of patients with stable coronary disease [6, 7]. The guidelines request objective evidence of ischemia in the perfusion bed of the coronary artery for which PCI is intended. Such proof of regional ischemia can also be provided by cardiac imaging. However, at inclusion into FAME 2 only slightly more than 20 % of patients had undergone any ischemia test. The situation in clinical practice may be even worse: many patients still undergo PCI without proof of regional ischemia and without use of FFR. The good outcome in patients with negative FFR which were followed-up in a separate registry in FAME 2 indicates that proof of ischemia is indeed mandatory before performing PCI in stable CAD patients.

## References

[1] De Bruyne B, Pijls NH, Kalesan B, Barbato E, Tonino PA, Piroth Z, Jagic N, Mobius-Winkler S, Rioufol G, Witt N, Kala P, MacCarthy P, Engstrom T, Oldroyd KG, Mavromatis K, Manoharan G, Verlee P, Frobert O, Curzen N, Johnson JB, Juni P, Fearon WF, Investigators FT (2012). Fractional flow reserve-guided PCI versus medical therapy in stable coronary disease. N Engl J Med.

[2] Redberg RF (2014). Sham controls in medical device trials. N Engl J Med.

[3] Bhatt DL, Kandzari DE, O’Neill WW, D’Agostino R, Flack JM, Katzen BT, Leon MB, Liu M, Mauri L, Negoita M, Cohen SA, Oparil S, Rocha-Singh K, Townsend RR, Bakris GL, Investigators SH (2014). A controlled trial of renal denervation for resistant hypertension. N Engl J Med.

[4] Campeau L (2002). The Canadian Cardiovascular Society grading of angina pectoris revisited 30 years later. Can J Cardiol.

[5] De Bruyne B, Fearon WF, Pijls NH, Barbato E, Tonino P, Piroth Z, Jagic N, Mobius-Winckler S, Rioufol G, Witt N, Kala P, MacCarthy P, Engstrom T, Oldroyd K, Mavromatis K, Manoharan G, Verlee P, Frobert O, Curzen N, Johnson JB, Limacher A, Nuesch E, Juni P, Investigators FT (2014). Fractional flow reserve-guided PCI for stable coronary artery disease. N Engl J Med.

[6] Task Force Members, Montalescot G, Sechtem U, Achenbach S, Andreotti F, Arden C, Budaj A, Bugiardini R, Crea F, Cuisset T, Di Mario C, Ferreira JR, Gersh BJ, Gitt AK, Hulot JS, Marx N, Opie LH, Pfisterer M, Prescott E, Ruschitzka F, Sabate M, Senior R, Taggart DP, van der Wall EE, Vrints CJ, Guidelines ESCCfP, Zamorano JL, Achenbach S, Baumgartner, H, Bax JJ, Bueno H, Dean V, Deaton C, Erol C, Fagard R, Ferrari R, Hasdai D, Hoes AW, Kirchhof P, Knuuti J, Kolh P, Lancellotti P, Linhart A, Nihoyannopoulos P, Piepoli MF, Ponikowski P, Sirnes PA, Tamargo JL, Tendera M, Torbicki A, Wijns W, Windecker S, Document R, Knuuti J, Valgimigli M, Bueno H, Claeys MJ, Donner-Banzhoff N, Erol C, Frank H, Funck-Brentano C, Gaemperli O, Gonzalez-Juanatey JR, Hamilos M, Hasdai D, Husted S, James SK, Kervinen K, Kolh P, Kristensen SD, Lancellotti P, Maggioni AP, Piepoli MF, Pries AR, Romeo F, Ryden L, Simoons ML, Sirnes PA, Steg PG, Timmis A, Wijns W, Windecker S, Yildirir A, Zamorano JL (2013) 2013 ESC guidelines on the management of stable coronary artery disease: The Task Force on the management of stable coronary artery disease of the European Society of Cardiology. Eur Heart J 34:2949–3003 10.1093/eurheartj/eht296 23996286

[7] Windecker S, Kolh P, Alfonso F, Collet JP, Cremer J, Falk V, Filippatos G, Hamm C, Head SJ, Juni P, Kappetein AP, Kastrati A, Knuuti J, Landmesser U, Laufer G, Neumann FJ, Richter DJ, Schauerte P, Sousa Uva M, Stefanini GG, Taggart DP, Torracca L, Valgimigli M, Wijns W, Witkowski A, Authors/Task Force Members, Authors/Task Force m (2014). 2014 ESC/EACTS Guidelines on myocardial revascularization: the Task Force on myocardial revascularization of the European Society of Cardiology (ESC) and the European Association for Cardio-Thoracic Surgery (EACTS)Developed with the special contribution of the European Association of Percutaneous Cardiovascular Interventions (EAPCI). Eur Heart J.

